# Adequate lung sliding identification is not influenced by the level of academic or ultrasound training

**DOI:** 10.1186/cc10692

**Published:** 2012-03-20

**Authors:** E Piette, R Daoust, J Lambert, A Denault

**Affiliations:** 1 Hôpital du Sacré-Coeur de Montréal, Canada; 2 Université de Montréal, Canada; 3 Institut de Cardiologie de Montréal, Canada

## Introduction

Rapid confirmation of the adequacy of endotracheal intubation is critical in the field of emergency medicine (EM). Methods confirming endotracheal tube (ET) position should have accuracy near 100%. Studies confirming ET position using lung sliding (LS) identification were done by physicians with extensive ultrasound (US) training using sometimes lengthy examination. These conditions are not easily reproduced in the emergency department. Our primary objective was to compare the accuracy of EM physicians with different levels of academic and US training to correctly identify presence or absence of LS on random short sequences of lung US. Our secondary objective was to determine if results were better when participants had the choice to abstain themselves in uncertain cases.

## Methods

We recorded in the operating room 280 short lung US sequences (one respiratory cycle), of present and absent LS of intubated patients and randomly presented them to two groups of EM physicians. Accuracy was calculated for different academic and US training: none, basic Focused Assessment with Sonography in Trauma (FAST), FAST and advanced cardiac US, fellowship in EM US. We compared them using an ANOVA test. Only participants in the second group where instructed to abstain from answering in uncertain cases and accuracy was compared to the first group using a Student's *t *test. The project was approved by the research and ethics committees.

## Results

Two medical students, 42 EM residents and 31 EM attendings participated. No difference in accuracy was shown between the subgroups of academic training with mean accuracies of 66.3% (medical students), 70.9% (residents) and 69.0% (attendings) (*P *= 0.361). No difference was shown between the subgroups of US training with means of 63.9% (no formation), 70.2% (FAST), 70.9% (FAST + advanced cardiac US), and 74.2% (fellowship) (*P *= 0.119). Accuracy was significantly better when participants could abstain from answering in uncertain cases with means of 67.5% (95% CI: 65.7 to 69.4) in the first group and 73.1% (95% CI: 70.7 to 75.5) in the second (*P *< 0.001).

## Conclusion

Correct LS identification on short lung US sequences is not influenced by the level of academic or US training. Accuracy is better when the possibility to abstain oneself from answering is given. LS identification using one respiratory US sequences should be used with caution to confirm adequacy of endotracheal intubation.

